# Normal seminal plasma could preserve human spermatozoa against cryopreservation damages in Oligozoospermic patients

**DOI:** 10.1186/s12860-021-00390-6

**Published:** 2021-10-05

**Authors:** Fatemeh Eini, Maryam Azizi kutenaei, Maryam Hosseinzadeh Shirzeyli, Zeinolabedin Sharifian Dastjerdi, Mahmoud Omidi, Marefat Ghaffari Novin

**Affiliations:** 1 Fertility and Infertility Research Center, Hormozgan University of Medical Sciences, Bandar Abbas, Iran; 2 Department of Biology and Anatomical Sciences, School of Medicine, Shahid Beheshti University of Medical Sciences, Tehran, Iran; 3 Department of Anatomical Sciences, Faculty of Medicine, Hormozgan University of Medical Sciences, Bandar Abbas, Iran; 4 Department of Pharmacology and Toxicology, Faculty of Pharmacy, Hormozgan University of Medical Sciences, Bandar Abbas, Iran; 5 Men’s health and Reproductive Health Research Center, Shahid Beheshti University of Medical Sciences, Tehran, Iran

**Keywords:** Cryopreservation, Human spermatozoa, Oligozoospermic patients, Seminal plasma

## Abstract

**Background:**

Cryopreservation of human spermatozoa has been identified as an efficient procedure to preserve fertility in men before any cancer therapy or surgical infertility treatment. Despite the benefits of the procedure, the deleterious effects of cryopreservation have been proven on sperm structure and function. This study aimed to evaluate seminal plasma effects on human sperm characteristics after cryopreservation, and compare the addition of normozoospermic and oligozoospermic seminal plasma in the prepared oligozoospermic samples. Semen samples were collected from fifty-five oligozoospermic men and the twenty fertile individuals who referred to the infertility center. At first, a semen analysis was carried out on each neat ejaculate, and then some were cryopreserved. The remainder of the semen was divided into two, one for seminal plasma removal and the other for sperm preparation. Then, the prepared spermatozoa were cryopreserved in three groups: one with, and another without the addition of oligozoospermic seminal plasma, and still another with the addition of normal seminal plasma. After thawing, sperm DNA integrity, viability, motility, and morphology were determined.

**Results:**

The percentages of all parameters were significantly lower after cryopreservation in all groups compared to the fresh sample. However, this reduction was lower in the oligozoospermic samples cryopreserved with normal seminal plasma.

**Conclusion:**

The results indicated that seminal plasma in oligozoospermic patients could not support sperm against cryo-injuries, an indication likely due to insufficient antioxidants and other protective components in oligozoospermic patients. However, normal seminal plasma could slightly preserve sperm characteristics after cryopreservation in oligozoospermic patients.

## Introduction

Human sperm cryopreservation is a helpful therapeutic approach to preserve fertility in several possible disorders [1]. Sperm cryopreservation is essential in patients undergoing chemotherapy or radiotherapy in malignancies or other surgical manipulations that may induce male reproductive dysfunctions [2]. Moreover, sperm cryopreservation is recommended in oligozoospermic patients at an increased risk of being azoospermic [3]. In such cases, sperm freezing could be a supportive approach to preserve fertility, and after sperm thawing, it can be used for assisted reproductive techniques (ART) [4].

Although spermatozoa cryopreservation was presented in the early 1960s and modified by various techniques, the effects of cryopreservation on sperm quality have not been desirable until now [3]. During cryopreservation, numerous factors including cold shock, ice crystal formation, and osmotic stress can cause damages to the chromatin configuration, organelle structure and morphological changes in the spermatozoa [5]. These factors are associated with the production of reactive oxygen species (ROS) [6]. Reactive oxygen species have been demonstrated to be attributed to the apoptosis, membrane lipid peroxidation, and DNA fragmentation [5].

Oxidative stress can be the main factor responsible for sperm DNA fragmentation. It has been shown that a high level of ROS is associated with DNA fragmentation in spermatozoa [7]. Subsequently, sperm DNA fragmentation is related to a low rate of fertilization and embryonic development. In general, cryopreservation has adverse effects on sperm functionality via affecting sperm motility, viability, morphology, and chromatin integrity and, thus, affecting the sperm fertilization potential [8, 9]. However, the functionality of post-thaw spermatozoa is predictable if seminal plasma compositions and spermatozoa quality are known before freezing [10].

Seminal plasma contains different ingredients including proteins, fatty acids, and minerals which serve as a medium to protect and nourish sperm up to fertilization [11]. Moreover, seminal plasma is a complex fluid with different antioxidant systems which protect sperm against ROS and DNA damages produced by cryopreservation or intra- or extra-cellular stressor conditions [10]. Also, there is an enzymatic protection system in seminal plasma fluid containing superoxide dismutase (SOD), glutathione peroxidase (GPx) and catalase and non-enzymatic antioxidants such as glutathione, ascorbic acid, vitamin E, Albumin and taurine [12, 13]. Moreover, polyunsaturated fatty acids play a crucial role in sperm viability and motility [10]. Besides, there is a relationship between fatty acid composition and the antioxidant capacity of seminal plasma. However, seminal plasma compositions change in different causes of infertility [14, 15], in which cases, Zalata et al. demonstrated that the fatty acid profile in fertile men is different from asthenozoospermic and oligozoospermic men [16]. The variations in the fatty acid profile and antioxidant capacity in seminal plasma and sperm functionality of different semen samples resulted in a variety of cryo-tolerance [17].

The high quality of pre-freezing semen parameters including sperm concentration, progressive motility, and morphology improve the potential cryo-tolerance of spermatozoa. In comparison with fertile men, oligozoospermic patients have a significantly lower cryotolerance of spermatozoa regarding seminal plasma compositions and sperm parameters. It seems that there are not enough fatty acids and antioxidant capacities to preserve sperm against cryo-injury damages in oligozoospermic patients [18]. Recent data suggest that oligozoospermic semen having higher levels of ROS can cause DNA damages to spermatozoa during cryopreservation [19, 20]. Thus, many studies indicated that adding antioxidants improves sperm parameters during semen cryopreservation [19–21].

In this study, we suggested that the seminal plasma composition could not support the spermatozoa against cryopreservation damages in oligozoospermic patients. Thus, the objective of our study was to evaluate whether seminal plasma in fertile men could improve cryotolerance of spermatozoa in oligozoospermic patients via analyzing viability, motility, morphology and DNA integrity of sperm during cryopreservation.

## Materials and methods

### Study design

This prospective study included fifty-five male partners from infertile couples with a sperm concentration of ≤15 × 10 ^6^ sperm/ml, who referred to the academic center of reproductive medicine in Taleghani Hospital. The study was approved by the Research Ethics Committee of Shahid Beheshti University of medical sciences (Approval No. SBMU.REC.1393.91). All patients were asked to sign a written letter of informed consent to take part in the study. The following exclusion criteria were used: abnormal sperm morphology with normal forms < 4%; semen volume < 2 mL; progressive motility< 32%; according to the World Health Organization (WHO) guidelines [22]. Moreover, males with leukocytospermia)leukocyte count ≥1 × 10^6^/mL(, or displaying any symptom or history of infection in the urogenital system were excluded due to the increased concentration of inflammatory cytokines or the possibility of seminal infection.

### Semen collection, division and analysis

**Fig. 1:**
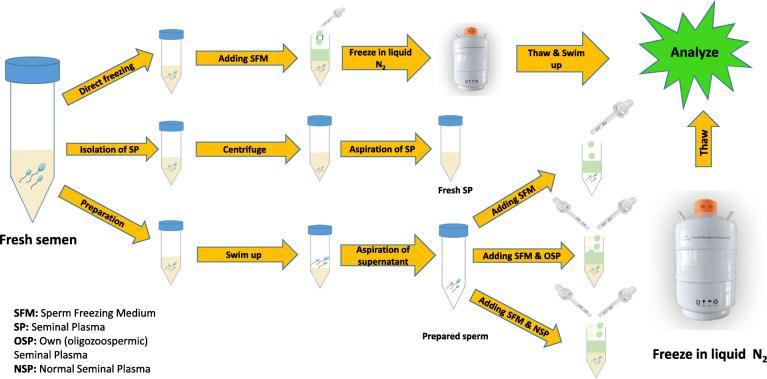
Study design

The samples were obtained from 25 mild and 30 moderate oligozoospermic patients, as defined by WHO (2010) criteria [22]. Moreover, to evaluate normal seminal plasma effect on sperm parameters during cryopreservation, 20 samples were collected from 20 normozoospermic men. The inclusion criteria for normozoospermic men were defined according to WHO criteria. The semen specimens were collected by masturbation into non-toxic sterile containers, following 3–5 days of ejaculatory abstinence. All semen samples were incubated for 30–45 min at 37 °C until total liquefaction. Semen analysis was carried out in all liquefied samples using the WHO guidelines [22] and then oligozoospermic samples were divided into three aliquots. One aliquot of neat semen was frozen unprepared. The second was processed for seminal plasma separation, as detailed below, and the third was prepared by a direct swim-up procedure. After the swim-up, once again, the prepared spermatozoa were analyzed for morphology, motility, vitality, and DNA fragmentation and then divided into three aliquots. Therefore, four groups of the same oligozoospermic samples were cryopreserved; neat semen, prepared semen without the addition of seminal plasma (SP-group), prepared sperm with addition of own (oligozoospermic) seminal plasma (OSP-group), and prepared sperm with the addition of normal seminal plasma (NSP-group). Also, normozoospermic ejaculates were used for normal seminal plasma separation (Fig. 1).

### Seminal plasma preparation

One of the aliquot of each oligozoospermic semen sample was centrifuged in a 1.5 mL microfuge tube at 400 g for 15 min. Then, the supernatant was aspirated and equal volume added to prepared oligozoospermic spermatozoa before cryopreservation. Also seminal plasma preparation was carried out for normozoospermic samples. Before cryopreservation, fresh normozoospermic seminal plasma was added to the oligozoospermic prepared sperm. Each normozoospermic seminal plasma was randomly used for more than one oligozoospermic prepared sperm. Therefore, the prepared oligozoospermic samples were cryopreserved with an equal volume of seminal plasma or without.

### Preparation of sperm by the direct swim-up procedure

Another aliquot of each semen sample was layered beneath an equal volume of Ham’s F10 medium containing 5% human serum albumin (HAS) in a sterile 10 ml conical based centrifuge tube. The tube was incubated for 1 h at 37 °C in 5% CO2 at an angle of 45° to increase the surface area in which the sperm could swim. The upper layer containing the motile sperm was then carefully removed and centrifuged at 300 g for 5 min. The supernatant was discarded and the pellet resuspended in 300 μl of Ham’s F10 medium to assess sperm quality before cryopreservation. Moreover, sperm concentration was standardized before cryopreservation.

### Motility assessment

Sperm motility was evaluated under phase contrast microscope using 400X magnification. The percentage of motile spermatozoa was evaluated according to WHO guidelines. Sperm motility was assessed based on a four-category scheme: rapid progressive, slow progressive, non-progressive and immotile). At least 200 sperms in at least five microscope fields of view were counted for each sample.

### Viability assessment

Eosin/Nigrosin staining was used for sperm viability assessment. Staining was performed using Eosine (1%)-and Nigrosin (10%) (EN; comprised of 0.2 g of eosin and 2 g of nigrosin dissolved in a 20 ml buffered saline solution [153 mM NaCl and 9.65 mM NaH2PO4; pH 7.4]). Equal volumes of sperm samples were added and then mixed and incubated for 30 s at room temperature. A smear was made on a glass slide and was allowed to dry. Unstained or light pink as live and red or dark pink colored as dead were evaluated at 1000x magnification under oil-immersion. Sperm viability was defined as the percentage of live cells. At least 200 sperms were counted for each sample.

### Morphology assessment

Sperm morphology was assessed in semen and the prepared sperm using strict criteria. 5 μl of the sample was smeared onto clean, glass slides and was allowed to air-dry for 20 min. The smears were stained by a diff-quick kit (Baxter Dade diagnostics AG, Dubingen, Switzerland). The morphology assessment was done according to WHO guidelines [22]. To evaluate sperm morphology, at least 100 sperms were counted at 1000x magnification under oil-immersion.

### DNA fragmentation

Sperm DNA integrity was determined by a Sperm DNA fragmentation assay kit (SDFA; ACECR, Tehran, Iran) according to manufacturer’s instructions [23]. Based on the sperm chromatin dispersion (SCD), five pattern were divided into two groups as follows: sperm with intact DNA: the spermatozoa with large- and medium-sized haloes; sperm with fragmented DNA: the spermatozoa with small-sized haloes or without a hallo or degraded spermatozoa [24]. A minimum of 200 spermatozoa per sample were scored under light microscopy at 1000x magnification. DNA fragmentation was defined as the percentage of spermatozoa with fragmented DNA.

### Cryopreservation and thawing procedure

Each aliquots was diluted 1:1 (v/v) with sperm freezing medium (LifeGlobal) which contained physiologic salts, glycine, dextrose monohydrate, lactate, glycerol, sucrose, and human serum albumin (3.95 mg/ml). Then 500 μL aliquots of each diluted sample were loaded into the straw and both ends were sealed using a heat sealer. The straws were frozen in exposure to liquid nitrogen (LN) vapor (5 cm above LN level) for 20 min. The straws were transferred into the tube and plunged into the LN. After three months, the straw was thawed in a 37 °C water bath. The two ends of the straws were cut and then the samples were poured into microtubes and 1/5 ml of pre-warmed medium (Ham’s F10 -HSA) passed through the straws. Then, the samples were centrifuged at 300 g for 5 min and the cells were resuspended in 100 μL of the medium. Finally, sperm suspensions were incubated at 37 °C for 10 min for further analysis.

### Statistical analysis

Statistical analysis was carried out using GraphPad Prism data analysis program (GraphPad Software, Inc., San Diego, CA, USA). In the present study, variables were expressed as mean ± SEM. Results from all sperm parameters were analyzed by either Student’s t-test (before and after cryopreservation in semen and prepared sperm) or one-way ANOVA followed by Tukey’s HSD post hoc test (comparison of sperm parameters between 4 groups of the study after cryopreservation). All statistical analyses were two-sided and *p* < 0.05 was considered as the statistically significant level.

## Results

### Effect of cryopreservation on Oligozoospermic semen parameters

As shown in Table 1, there was a significant reduction in the percentage of sperm motility, morphology, and viability after semen cryopreservation compared to fresh semen samples. Moreover, regarding DNA fragmentation, a significant increase was observed in cryopreserved semen in comparison with the fresh samples (*p* < 0.001).

**Table 1:** Effect of cryopreservation on sperm parameters in oligozoospermic neat semen samples

Neat semen parameters	Before cryopreservation (***N*** = 55)	After cryopreservation (N = 55)	***P*** < 0.001	***Cry-thaw Prepared semen***
**Sperm volume (mL)**	3.95 ± 1.6	–	–	–
**Sperm count (10**^**6**^**/mL)**	10.65 ± 2.88	9.21 ± 1.48	–	8.23 ± 1.65
**Viability %**	52.24 ± 1.71	19.97 ± 1.19	***	28.94 ± 0.98
**Normal morphology %**	4.03 ± 0.45	1.67 ± 0.30	***	2.35 ± 0.43
**Motility %**	24.70 ± 1.83	9.70 ± 0.88	***	19.00 ± 1.42
**DNA fragmentation %**	37.94 ± 3.06	62.03 ± 3.31	***	52.46 ± 4.67

Data are represented as mean ± SEM

Student t-test: values between sperm parameters before and after cryopreservation; ***: significant at *P* < 0.001

**Fig. 2:**
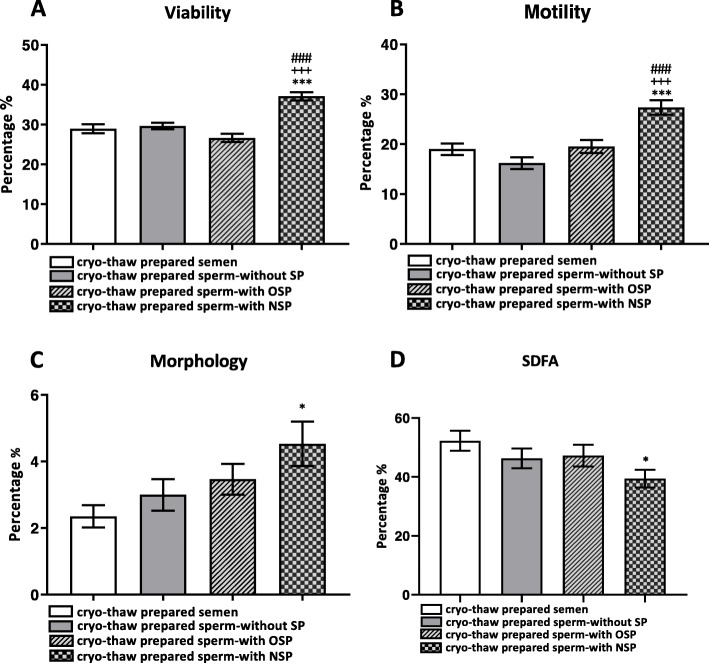
Effect of cryopreservation on sperm parameters of oligozoospermic samples in four different groups. A. Sperm viability, B. Sperm motility, C. Sperm morphology, and D. Sperm DNA fragmentation. *P < 0.05 and ***P < 0.001 versus the cryo-thaw prepared semen, ^+++^P < 0.001 versus the cryo-thaw prepared sperm without seminal plasma (SP), ^###^P < 0.001 versus the cryo-thaw prepared sperm with oligozoospermic seminal plasma (OSP) by one-way ANOVA. NSP: Normozoospermic seminal plasma

After thawing, the preparation of cryopreserved semen samples through the swim up-procedure increased the percentage of sperm parameters, including motility, viability, and morphology and DNA integrity (Table 1 and Fig. 2: cryo-thaw prepared semen). However, these values were significantly lower than fresh prepared semen with regard to decreases in sperm count after the swim up (Table 2).

**Table 2:** Effect of cryopreservation on sperm parameters in oligozoospermic prepared semen samples

Prepared semen parameters	Before cryopreservation (N = 55)	After cryopreservation (N = 55)	***P*** < 0.001
**Sperm volume (mL)**	3.95 ± 1.6	–	
**Sperm count (10**^**6**^**/ mL)**	4.31 ± 1.58	3.68 ± 1.20	–
**Viability %**	64.53 ± 2.17	29.65 ± 1.12	***
**Normal morphology %**	7.06 ± 0.96	3.00 ± 0.64	***
**Motility %**	53.00 ± 3.18	16.21 ± 1.78	***
**DNA fragmentation %**	26.12 ± 2.81	46.29 ± 4.91	***

Data are represented as mean ± SEM. t-test values; ***: significant at P < 0.001

### Effect of seminal plasma on sperm viability during cryopreservation

The data concerning sperm viability are presented in Fig. 2A. Adding oligozoospermic seminal plasma to the prepared sperm did not enhance sperm viability prior to cryopreservation. However, viability percentage was improved in the prepared sperms by supplementing normal seminal plasma in comparison with other groups (NSP: 37.12 ± 1.43 vs. (OSP: 26.65 ± 1.28; *p* < 0.001).

### Effect of seminal plasma on sperm motility during cryopreservation

It was established that, in sperm motility rate, there was no significant difference between the cryopreservation of neat semen and the prepared sperm. Moreover, adding seminal plasma to prepared sperm did not account for any significant difference in motility rate compared to the prepared sperm without adding any seminal plasma. However, a significant increase was observed in motility rate in the prepared sperm which were frozen by adding normal seminal plasma prior to the cryopreservation (NSP: 27.35 ± 1.81 vs. OSP: 19.53 ± 1.39; *p* < 0.001) (Fig. 2B).

### Effect of seminal plasma on sperm morphology during cryopreservation

As shown in Fig. 2C, supplementing the prepared sperm with oligozoospermic seminal plasma did not have any significant effect on morphology rate. Oligozoospermic semen did not preserve sperm structure against the deteriorative effect of cryopreservation in either the neat or the prepared sperm. However, a significant increase was observed in morphology of prepared sperms by adding normal seminal plasma prior to cryopreservation when compared to the cryo-thaw prepared semen (NSP: 4.53 ± 0.73 vs. OSP: 3.47 ± 0.66; *p* < 0.001).

### Effect of seminal plasma on sperm DNA integrity during cryopreservation

Oligozoospermic seminal plasma did not preserve sperm DNA integrity against the adverse effects of cryopreservation. Moreover, the cryopreservation of prepared sperms without adding the oligozoospermic seminal plasma had similar effects on DNA integrity. However, DNA integrity was improved when normal seminal plasma was added to the prepared sperm before cryopreservation in comparison to the cryo-thaw prepared semen (NSP: 39.41 ± 4.78 vs. OSP: 47.21 ± 4.39; *p* < 0.001) (Fig. 2D).

## Discussion

In this study, we evaluated the addition of normal seminal plasma to oligozoospermic samples to investigate its effect on sperm parameters and DNA fragmentation during cryopreservation. Since the oligozoospermic seminal plasma has a different composition from the normal one and can adversely affect sperm parameters, cryopreservation was carried out for four sperm preparation groups, namely, neat semen from an oligozoospermic ejaculate, prepared sperm from an oligozoospermic ejaculate without adding seminal plasma, prepared sperm from the oligozoospermic ejaculate with adding oligozoospermic seminal plasma, and prepared sperm from the oligozoospermic ejaculate with adding normal seminal plasma. Our results indicated no effect of oligozoospermic seminal plasma on sperm parameters. Seminal plasma from oligozoospermic ejaculates did not keep spermatozoa from the detrimental effects of cryopreservation. However, the seminal plasma from normozoospermic men could preserve spermatozoa from the adverse effects of cryopreservation.

Our results showed that a beneficial procedure for cryopreservation of oligozoospermic sample is to freeze the prepared sperm in normal seminal plasma. Also, there were no differences in sperm parameters between neat semen and prepared sperm in oligozoospermic patients during cryopreservation. These results showed that the seminal plasma in oligozoospermic patients did not have any supportive effect during cryopreservation.

Spermatozoa loses a large amount of its cytoplasm during spermatogenesis. Therefore, the spermatozoa does not have any functional endogenous antioxidant system against ROS production [25]. However, seminal plasma contains several antioxidants and scavengers which preserve spermatozoa from ROS damages. Also, it contains enzymatic antioxidants including superoxide dismutase and catalase and some scavengers including albumin and taurine. However, a number of studies have shown that seminal plasma in sub-fertile and infertile men, due to the reduction of antioxidants, could cause spermatozoa to be very vulnerable to cryopreservation-induced stress [10, 15]. There is research evidence that the lack of antioxidants in seminal plasma could have deteriorative effects on sperm DNA integrity [26]. In sub-fertile and infertile semen samples, sperm chromatin integrity is poorly organized and sensitized to the induced damage by cryopreservation in contrast to fertile men [27]. Moreover, due to the protamine packaging protective property of sperm DNA against ROS damages, insufficient protamination in oligozoospermic men makes sperm chromatin susceptible to oxidative stress injuries [28]. Sperm chromatin damages or DNA denaturation are related to the impairment of fertilization abilities and embryo development. Therefore, recent investigations focus on the modification of sperm cryopreservation methods to prevent sperm DNA damages during freezing [29].

Seminal plasma and its composition play a crucial role in the quality of semen samples with a low and rare count of spermatozoa. The deficiencies of some components, including proteins and non-enzymatic and enzymatic antioxidants, have been reported in severe oligozoospermic and azoospermic individuals [3]. These components play a major role in preserving low numbers of spermatozoa against freezing damages. Potts et al. (2000) reported that adding seminal plasma to the culture medium of spermatozoa could reduce exogenous oxidative stress [30]. Consistent with this finding, our results suggested that adding normozoospermic seminal plasma to oligozoospermic prepared sperm reduced the DNA fragmentation induced by oxidative stress during the freezing procedure. In the study by Blanco et al. (2008), the results showed that the high seminal plasma antioxidant activity positively correlated with high semen quality and increase in sperm DNA integrity [31]. Moreover, the proteins in the seminal plasma, clearly insufficient in oligozoospermic patients, have critical roles to preserve sperm from cryopreservation damages [25]. Therefore, in our results, DNA integrity in group NSP is high due to the addition of normozoospermic seminal plasma to prepared oligozoospermic samples in this group. We also found that, adding oligozoospermic seminal plasma to prepared spermatozoa has no effect on DNA fragmentation before cryopreservation. Therefore, the results of sperm DNA integrity were not different in two groups: cryopreservation of prepared spermatozoa with or without oligozoospermic seminal plasma. These results are compatible with previous studies showing that the cryopreservation induces sperm DNA fragmentation in normozoospermic and oligozoospermic patients. However, it is noteworthy that regardless of the poor quality seminal plasma in oligozoospermic patients, the spermatozoa from these semen samples were highly vulnerable to cryopreservation damages because of the poor chromatin configuration [4].

Cryopreservation has been shown to reduce the progressive motility and viability of spermatozoa for approximately more than 35% in both fertile and infertile patients. This reduction is higher in sub-fertile and infertile patients because of a more vulnerability of spermatozoa to osmotic and oxidative stresses following freezing [2]. In this study, we showed that the motility and viability rates were higher in prepared spermatozoa with the addition of normozoospermic seminal plasma compared to the other groups after cryopreservation. The reduction of motility and viability after cryopreservation in neat semen or prepared sperm indicated that seminal plasma did not have any protective effect against cryopreservation injures in oligozoospermic patients. However, the cryo-thaw sperm with the addition of normal seminal plasma exhibited more motility and viability rates after cryopreservation in comparison with other groups. The results of this study are in agreement with previous studies regarding the loss of sperm motility and viability in oligozoospermic patients during cryopreservation [32].

Cryopreservation also leads to a decrease in sperm morphology rate [33]. It has been reported that cryopreserved spermatozoa suffered a significant reduction in morphology rate with high defects in the head, midpiece and tail [34]. Osmotic stress has deteriorative effects on sperm morphology via ice crystal formation, cellular dehydration and cell shrinkage which result in permanent damages [35]. The four groups in this study showed a reduction in morphology rate following cryopreservation. The decreases were similar in three groups except for the fourth group, with a lower reduction than others. Therefore, compared to other groups in which no morphology preservation was observed, normozoospermic seminal plasma could slightly preserve sperm morphology against cryopreservation damages.

In conclusion, our results showed that the rare and low count of sperm in oligozoospermic patients are very vulnerable to cryo-injuries. On the other hand, in these patients, the low quality of seminal plasma could not preserve spermatozoa against cryopreservation damages with respect to the possibly insufficient antioxidant capacity and other supplements including proteins and fatty acids. However, the addition of normozoospermic seminal plasma to the prepared spermatozoa from oligozoospermic patients has beneficial effects on sperm motility, viability, morphology, and DNA integrity against cryopreservation.

## Data Availability

The datasets used and/or analyzed during the current study are available from the corresponding author on reasonable request.
